# Brunetti’s Method of Preparing and Preserving Anatomical Specimens

**Published:** 1868-03

**Authors:** 


### BRUNETTI'S METHOD OF PREPARING AND PRESERVING ANATOMICAL SPECIMENS.

The Paris correspondent of the Vienna "*Med. Presse*" (Sept. 1, 1867) speaks in the highest terms of praise of Prof. Brunetti's important invention of a mode of preparing anatomical specimens of great durability, which will exert a large influence upon the respective branches of medical study and education. The principal characteristic of Brunetti's preparations, says the correspondent, is certainly their astounding lightness; their color is usually light-grey or white; [the first trials of the method produced darker, more brownish specimens]. All the membranous formations and walls have acquired such a degree of dryness and rigidity that all preparations are preserved in their primitive form and in their individual topographical relations—one of those circumstances which prove and secure their scientific value. All bloodvessels, very distinctly visible in sections, are wide open; all, even the smallest and finest distributions of vessels, as well as the pulmonary vesicles, are clearly and prettily defined. The uriniferous tubes of the kidney and the glandular orifices in all parts of the intestinal canal, etc., etc., can be observed with facility, and thus an accurate image can be obtained, with a magnifying power of only 40-60, of the minute composition and intimate structure of organs. If a higher power is to be used, however, the objects should be rendered transparent by imbibition of water or glycerine, and as strongly illuminated by transmitted light as possible. . . . . .

What more especially facilitates the microscopical examination of the oldest, very dry preparations, is the fact that they are most excellently adapted to the cutting of very fine sections, and pretty large sections can thus be made without too great difficulty. No other sort of recent or preserved anatomical specimens affords this advantage in so great a degree; so that it is probable that by the aid of Brunetti's preparations the study of the architectonic composition and arrangement of the individual anatomical elements of each organ can be essentially advanced and brought to greater perfection.

Even in the study of topographical anatomy these preparations may be of much use, as all parts are mummified *living*, as it were, and most of the characteristics pertaining to them during life are preserved. Sections can be made in any direction, at all points, and the cut surfaces remain unaltered for an indefinite time. Moreover, all preparations from whatever region or organ are completely devoid of odor. . . . Excepting a very few fine and delicate specimens, they need not be handled with very great care, and most of them are sufficiently protected from breakage or other injurious influences, as pressure, blows, falls, etc., by their elasticity.

A Paris letter of the *N. Y. Times* (*N. Y. Medical Journal*, Oct. 1867) says, in reference to this invention: Here are specimens of healthy and diseased liver, of healthy and diseased lung, of healthy and diseased kidney—in fact, of healthy and diseased tissue from all parts of the body. In the lung we see specimens of interstitial granulations, of tubercles, and of cavaties after abscesses. All this is so plain that a child might learn how the lung looks in the various diseased states to which it is subject. So, too, there are slices of liver and kidney showing fatty degeneration and cirrhosis of the first, and Bright's disease of the latter.

The process of Dr. Brunetti comprises several operations—*viz.*: 1, the washing of the piece to be preserved; 2, the *degraissage*, or eating away of the fatty matter; 3, the tanning; and 4, the desiccation.

1. To wash the piece M. Brunetti passes a current of pure water through the bloodvessels, and the various excretory canals, and then he washes the water out by a current of alcohol.

2. For destroying the fat he follows the alcohol with ether, which he pushes, of course, through the same bloodvessels and excretory ducts. This part of the operation lasts some hours. The ether penetrates the interstices of the flesh, and dissolves all the fat. The piece, at this point of the process, may be preserved any length of time desired, plunged in ether, before proceeding to the final operations.

3. For the tanning process M. Brunetti dissolves tannin in boiling distilled water, and then, after washing the ether out of the vessels with distilled water, he throws this solution in.

4. For the drying process, Dr. Brunetti places the pieces in a vase with a double bottom filled with boiling water, and he fills the places of the preceding liquids with warm, dry air. By the aid of a reservoir, in which air is compressed to about two atmospheres, and which communicates by a stop-cock and a system of tubes, first to a vase containing chloride of calcium, then with another heated, then with the vessels and excretory ducts of the anatomical piece in course of preparation, he establishes a gaseous current which expels in a very little time all the fluids. The operation is now finished.

The piece remains supple, light, preserves its size, its normal relations, its solid histological elements, for there are no longer any fluids in it. It may be handled without fear, and will last indefinitely. A similar, more easily executed, but probably much less advantageous process has been invented by M. Von Vetter, and is published in the *Chemical News* (in *Dental Cosmos*, Nov. 27): Add to 7 parts of glycerine at 22 deg. 1 part of raw brown sugar and half a part of nitre, till a slight deposit is formed at the bottom of the vessel. The portion required to be preserved is then plunged, dried or not dried, and left in the mixture for a time proportional to its dimensions; a hand, for example, should remain eight days in the liquid; when it is taken out it is as stiff as a piece of wood, but if it be suspended in a dry and warm place the muscles and articulation recover their suppleness.—*St. Louis Med. & Surg. Journal.*

